# Assessment of a Reduced SNP Panel Targeting Prolificacy and Coat Color Genes in Brazilian Sheep Breeds

**DOI:** 10.3390/ani16132008

**Published:** 2026-07-01

**Authors:** Camila Souza Rodrigues, Danielle Assis de Faria, Hymerson Costa Azevedo, Kleibe de Moraes Silva, Olivardo Facó, Sandra Aparecida Santos, Ramayana Menezes Braga, Alexandre Rodrigues Caetano, José Carlos Ferrugem Moraes, Carlos José Hoff de Souza, Samuel Rezende Paiva, Concepta McManus

**Affiliations:** 1 Faculty of Agronomy and Veterinary Medicine, Central Institute of Sciences, Darcy Ribeiro Campus, University of Brasília, Brasília 70910-900, DF, Brazil; rodriguesscamila@outlook.com; 2 Embrapa Recursos Genéticos e Biotecnologia, Brasília 70770-917, DF, Brazil; alexandre.caetano@embrapa.br (A.R.C.); samuel.paiva@embrapa.br (S.R.P.); 3 Embrapa Tabuleiros Costeiros, Aracaju 49025-040, SE, Brazil; hymerson.azevedo@embrapa.br; 4 Embrapa Caprinos e Ovinos, Sobral 62010-970, CE, Brazil; kleibe.silva@embrapa.br (K.d.M.S.); olivardo.faco@embrapa.br (O.F.); 5 Embrapa Pecuária Sudeste, São Carlos 13560-970, SP, Brazil; sandra.santos@embrapa.br; 6 Embrapa Roraima, Boa Vista 69301-970, RR, Brazil; ramayana.braga@embrapa.br; 7 Embrapa Pecuária Sul, Bagé 96401-970, RS, Brazil; jose.ferrugem-moraes@embrapa.br (J.C.F.M.); carlos.hoff-souza@embrapa.br (C.J.H.d.S.); 8 Center for Nuclear Energy in Agriculture, University of São Paulo, Piracicaba 13400-970, SP, Brazil; concepta@unb.br

**Keywords:** locally adapted sheep breeds, genetic diversity, candidate genes for production traits, genetic resources, germplasm bank

## Abstract

Prolificacy and coat color are key economic traits influencing sheep production. This study evaluated a reduced panel of 48 SNPs previously associated with these traits and assessed its applicability to locally adapted Brazilian sheep breeds. A total of 1152 samples from 15 breeds, conserved in situ at conservation nuclei and ex situ in the Brazilian Animal Germplasm Bank, were genotyped. Allelic, genotypic, and haplotypic frequencies were estimated to compare genetic variability between in situ and ex situ populations. Additionally, linkage disequilibrium (LD) among SNPs within *GDF9*, a key gene associated with prolificacy, was evaluated across local breeds, revealing strong LD among specific markers. The results highlight the importance of the FecG^E^ (*GDF9*) variant for prolificacy in hair sheep, confirming the presence of mutant allele E in prolific breeds, such as Santa Inês and Morada Nova, and identifying, for the first time, a high frequency of such allele in the Brazilian Blackbelly, which provides new insights into the genetic basis of this prolific hair breed. Other prolificacy-related genes, *BMP15* and *BMPR1B*, appear to have no functional role in locally adapted breeds, as initially hypothesized, considering genetic differences among European and tropical sheep. Allelic and genotypic variation in *ASIP*, *MC1R*, *TYRP1*, and *MITF* genes reflected differences between wool and hair sheep and between local and commercial breeds. Overall, the results indicate that the germplasm bank effectively preserves in situ diversity. In conclusion, the reduced SNP panel efficiently genotyped Brazilian sheep for prolificacy and coat color SNPs, confirming which markers are present and segregating in these breeds. However, its utility could be improved by removing markers of limited relevance in the targeted breeds.

## Simple Summary

Sheep play an important role in food production and rural livelihoods, especially in regions where locally adapted breeds have developed unique traits. Two key characteristics that influence sheep productivity are the number of lambs born per ewe (prolificacy) and coat color, which can affect adaptation and market value. This study evaluated genetic variations in 15 Brazilian sheep breeds using a set of 48 genetic markers related to these traits. Our findings suggest that a specific mutation in the *GDF9* gene may be associated with increased lamb production in the Brazilian Blackbelly, in addition to Santa Inês and Morada Nova breeds where it has previously been associated with prolificacy. Other genes formerly linked with enhanced reproductive performance did not show relevant effects in these locally adapted breeds. Genetic differences were also identified in genes influencing coat color, helping to explain the diversity observed between hair and wool sheep. Importantly, the genetic material stored in the Brazilian Animal Germplasm Bank (BBGA) preserves much of the diversity found in populations conserved in situ. These findings contribute to the conservation and sustainable use of local sheep breeds, supporting genetic improvement programs and helping ensure food security and biodiversity for future generations.

## 1. Introduction

Prolificacy and coat color are economically and adaptively important traits in sheep production. While prolificacy enhances productivity rates, coat color influences thermoregulation, breed characterization, and market preferences. Numerous single-nucleotide polymorphisms (SNPs) within key genes associated with these traits have been identified in a variety of commercial sheep breeds [1,2,3,4,5,6,7,8], particularly of European, North American, and Asian origin [9,10]. However, SNPs identified in these breeds may not always apply to locally adapted animals from tropical regions, such as Brazil, primarily due to differences in genetic background, selection history, and allele frequencies [11].

Reproductive performance in sheep is determined by both fecundity-related factors (e.g., ovulation rate and litter size) and fertility traits, including oocyte quality, follicular maturation, fertilization success, and embryo survival [12]. While prolificacy genes such as *GDF9*, *BMP15*, and *BMPR1B* can increase ovulation rate, certain allelic combinations, particularly homozygotes, may result in subfertility or sterility [1,2,3,4]. Therefore, selection for high litter size must account for potential trade-offs in overall fertility. Previously, the FecG^E^ (*GDF9*) variant has been associated with increased prolificacy in Santa Inês and Morada Nova, two of the most important hair sheep breeds in Brazil [13,14]. However, in *BMP15* and *BMPR1B*, mutations known to enhance prolificacy in several commercial breeds are absent or not associated with increased litter size in certain autochthonous breeds, including Santa Inês and Morada Nova [15].

Color-related loci influence not only sheep coat pigmentation but also breed authenticity, making them strategic markers for investigating genetic diversity in locally adapted breeds [16]. In the Brazilian Crioula, a local Brazilian breed, animals display a wide range of colors, with previous studies showing that epistatic interactions between specific alleles of the *MC1R* and *ASIP* genes contribute significantly to the observed coat color variation [17,18]. This historically important breed produces medullated wool that is primarily used for handicrafts [19]. Although not known for high-quality wool production, the breed is included in the conservation program of the Brazilian Agricultural Research Corporation (Embrapa), which maintains the main registered herd, as detailed in its 2011 phenotypic description [20].

Among Brazilian hair breeds, Santa Inês and Morada Nova are well adapted to the semi-arid and other tropical and subtropical conditions of the country, underscoring the importance of thermoregulation as a key adaptive trait in these breeds. Santa Inês exhibits a wide range of coat colors, from white to black, including brown and spotted, but white-coated individuals demonstrate greater heat tolerance [21]. Similarly, in Morada Nova, white and red individuals are officially recognized by the Brazilian Sheep Breeders Association (ARCO), but the white variety shows enhanced thermoregulatory capacity [22]. These findings highlight the genetic complexity of coat color in Brazilian sheep, shaped by selective pressures related to climate adaptation.

Despite presenting low production parameters compared to commercial breeds, locally adapted Brazilian sheep, especially Santa Inês, Morada Nova, and Crioula, are key genetic resources defined by resilience to harsh tropical production conditions and distinctive phenotypes [23]. However, these populations remain underrepresented in genomic studies. Additionally, current large-scale genotyping panels, mostly designed for commercial breeds, often prove financially unsustainable or overly complex for routine applications in smaller, in situ conservation and breeding programs involving local breeds. Therefore, this study addresses this gap by evaluating a reduced panel of 48 SNPs, previously associated with prolificacy and coat color, to determine which markers are present and segregating in locally adapted Brazilian sheep breeds. Given that these breeds are predominantly hair sheep and genetically distinct from European commercial breeds, we hypothesized that several alleles commonly reported in wool breeds may not segregate in Brazilian populations. Thus, the study primarily sought to identify the applicability of these SNPs in local breeds. Genotyping was conducted using a rapid genotyping methodology, which, if effective, can efficiently and cost-effectively support conservation and breeding programs by enabling the detection of allelic diversity and reducing reliance on monomorphic loci that cannot discriminate among breeds.

## 2. Materials and Methods

### 2.1. Biological Samples

A total of 1.040 whole blood samples from sheep of 15 breeds were accessed from the DNA and tissues bank of the Embrapa Genetic Resources and Biotechnology, located in Brasília, Brazil. These samples are from animals maintained at Embrapa’s in situ conservation centers distributed across the country. The breeds included in the study were Santa Inês, Morada Nova, Crioula, Brazilian Fat-tail, Brazilian Somali, Brazilian Bergamasca, Corriedale, Ile de France, Pantaneiro, Dorper, Damara, Suffolk, Hampshire, and Texel. Semen samples (N = 112) conserved ex situ at the Brazilian Animal Germplasm Bank (BBGA) were also included in the study. Breed descriptions and final sample sizes per population (after quality control) are presented in Table 1.

DNA extraction was performed using the Gentra Puregene Blood Kit (Qiagen, Germantown, MD, USA; https://www.qiagen.com/us (accessed on 27 November 2025)) for both whole blood and semen samples. The DNA quality of the samples was assessed in two ways. Initially, a 1% agarose gel stained with ethidium bromide was used, with lambda standards of 200 ng/µg, 100 ng/µg, and 50 ng/µg for comparison. Additionally, the samples were analyzed on a NanoDrop Thermo Scientific spectrophotometer (NanoDrop™ 8000, Thermo Fisher Scientific, Waltham, MA, USA; https://www.thermofisher.com/ (accessed on 27 November 2025)) to determine the presence of contaminants (proteins and chemical reagents).

### 2.2. Genotyping Methodology

The Fluidigm EP1™ system (Fluidigm Corporation, South San Francisco, CA, USA) was selected as the genotyping method for this study. Fluidigm^®^ SNP Type™ assays were designed for 35 prolificacy-related and 13 coat color-related pre-selected markers using the Fluidigm D3™ tool, following instructions and guidelines of the manufacturer, available at https://d3.fluidigm.com/ (accessed on 27 November 2025). Detailed information on the sequences of each marker used in the study is provided in Table S1. Each DNA sample had a total of 90 ng used for genotyping, and the data was generated following the standard protocols provided by Fluidigm for use with the EP1 platform. The samples underwent pre-amplification utilizing locus-specific primers (LSP) oligonucleotides and specific target amplification (STA) primers for the tested SNPs. The diluted amplified product was loaded onto twelve 96.96 Dynamic Array™ IFCs (Fluidigm Corporation, South San Francisco, CA, USA), with ROX reference dye and assays containing allele-specific primers (ASP) and LSP oligonucleotides for each SNP, according to the manufacturer’s instructions. Fluorescence endpoint image data were acquired on the EP1 Fluidigm imager, and genotype calls were obtained using the Fluidigm SNP Genotyping Analysis Software v4.1.3 (Fluidigm Corporation, South San Francisco, CA, USA).

### 2.3. Data Analysis

After performing cluster analysis to define genotype classes for each SNP, the confidence threshold for each genotype was set at >0.90. Samples and markers underwent quality control using SNP & Variation Suite (SVS) v8.9.1 (Golden Helix, Bozeman, MT, USA; https://www.goldenhelix.com/ (accessed on 27 November 2025)). Samples with a call rate < 0.80 and markers with a call rate < 0.75 were eliminated. Allelic, genotypic, and haplotypic frequencies were estimated using GenAlEx 6.5 [24], Arlequin 3.5.2.2 [25], and SHEsis web-based tool, available at http://analysis.bio-x.cn (accessed on 27 November 2025) [26,27], respectively. Monomorphic markers were excluded from the haplotype analysis. Linkage Disequilibrium (LD) was assessed using Haploview v.4.2, calculating D’ and r2 [28].

The potential association between prolificacy and coat color was assessed at the individual level in five major locally adapted hair sheep breeds: Brazilian Blackbelly, Morada Nova, Brazilian Fat-tail, Santa Inês, and Brazilian Somali, using a total of 476 individuals, with Pearson’s chi-squared test of independence [29]. Three markers were used: prolificacy-associated FecG^E^ (*GDF9*), and coat-color-associated *MC1R* p.M73K A>T and p.D121N A>G, for which 3 × 3 contingency tables were constructed based on the observed genotypes. Genotypes were numerically encoded as 1 = homozygous wild type, 2 = heterozygous, and 3 = homozygous mutant, and all analyses were performed using R statistical software, version 4.5.2 (R Core Team, Vienna, Austria, 2025) [30]. Because some contingency table cells contained low or zero expected counts, *p*-values were computed using Monte Carlo simulation with 10,000 replicates, implemented via the chisq.test() base R function. Bonferroni correction was applied across the three pairwise comparisons. Adjusted *p*-values were compared against α = 0.0167 (0.05/3).

## 3. Results

### 3.1. Quality Control

After quality control, 845 (73%) samples remained, out of the initial 1152. Due to the small sample sizes (≤5), in situ samples of Brazilian Bergamasca, Dorper, Hampshire, Brazilian Somali, and Texel were not included in the further analyses. Nonetheless, despite the limited number of samples, these breeds were genotyped to fulfill the study’s objective of covering the complete germplasm bank. From the 35 prolificacy-related markers, 12 (34%) were retained, as nine (26%) presented a call rate < 0.75, and 14 (40%) were monomorphic, and therefore excluded from further analysis. Of the 13 markers genotyped for coat color, eight (61%) were analyzed, as four (31%) were excluded by call rate (<0.75) and one marker (8%), the D_9_ indel (*ASIP*), was monomorphic, with all animals carrying the insertion variant (N) of the 9-bp fragment (AGCCGCCTC).

### 3.2. Prolificacy-Related SNPs

In the genes *BMP15* (chromosome X: 54,283,635–54,290,315; complement strand, ovine reference genome ARS-UI_Ramb_v3.0, NC_056080.1) and *BMPR1B* (chromosome 6: 30,028,547–30,482,585; complement strand, ARS-UI_Ramb_v3.0, NC_056059.1), most SNPs were excluded during quality control. At the B1 mutation, located in exon 1 of *BMP15*, the deletion variant was the most frequent in eight of the 15 breeds studied; however, all breeds also carried the CTT insertion, with the lowest frequency observed in Damara (15%) (Table 2). At the G192A variant in exon 2 of *BMPR1B*, the C allele was the most frequent in all breeds, fixed in ten and nearly fixed in the remaining five; the highest frequency of the T allele was found in the Pantaneiro (7%) (Table 2). The *GDF9* gene (chromosome 5: 42,113,878–42,116,604; complement strand; ARS-UI_Ramb_v3.0; NC_056058.1) had the highest number of markers that passed quality control, with 10 markers analyzed (Table 2). Genotypic frequencies of markers located in *BMP15*, *BMPR1B*, and *GDF9* are available in Table S2.

At the FecG^E^ variant, located in exon 2 of *GDF9*, the mutant allele E, previously associated with a high rate of twin births in sheep, was more frequent in the Brazilian Blackbelly (76%) and Morada Nova (71%) breeds. Allele E was also present in Brazilian Bergamasca (13%), Pantaneiro (7%), Brazilian Fat-tail (43%), Santa Inês (31%), and Brazilian Somali (30%) (Table 2). Notably, wild-type allele + is fixed in most wool breeds, such as Corriedale, Crioula, Ile de France, Hampshire, Suffolk, and Texel (Table 2).

**Figure 1:**
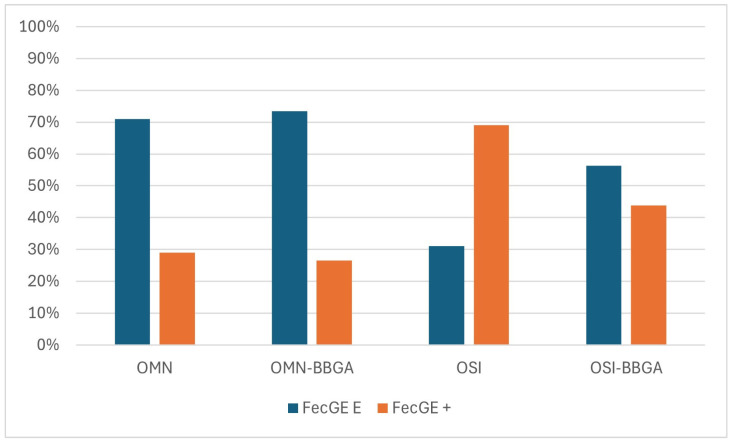
Comparison of allelic frequencies (%) of the FecG^E^ (*GDF9*) between the two most important Brazilian hair sheep genetic resources, conserved in situ at Embrapa’s conservation nuclei and ex situ at the Brazilian Animal Germplasm Bank (BBGA). OMN = Morada Nova (N = 129); OMN-BBGA (N = 32); OSI = Santa Inês (N = 233); OSI-BBGA (N = 16).

Comparison of Morada Nova and Santa Inês samples conserved in situ versus ex situ revealed that the BBGA germplasm closely resembled the conservation nuclei, with the FecG^E^ mutant allele present in both hair breeds (Figure 1). In Morada Nova, the mutant allele E was found at similarly high frequencies, slightly above 70%, in both populations. In contrast, Santa Inês from the BBGA exhibited a higher frequency of the FecG^E^ allele (56%) compared with the in situ conserved samples (31%), indicating a potential enrichment of the mutant allele in the ex situ germplasm collection.

### 3.3. Linkage Disequilibrium Analysis of GDF9

Using the D’ and r^2^ statistics, linkage disequilibrium (LD) among 10 *GDF9* markers (G1, G3, 3655, G4, 3776, FecG^E^, FecG^EII^, FecG^EI^, rs425223128, rs40376560) was assessed for each locally adapted breed separately. LD among the analyzed SNPs varied across breeds but consistently highlighted regions of strong allelic association, especially between FecG^EI^ and FecG^EII^ and between rs40376560 and FecG^E^ across most breeds, as values of r^2^ > 0.33 are in LD [31]. In the Brazilian Blackbelly and Brazilian Bergamasca, most SNP pairs exhibited high D’ values, indicating strong historical LD, with the highest r^2^ observed between rs40376560 and FecG^E^ (r^2^ = 0.88), suggesting this pair as highly predictive. Morada Nova and Santa Inês exhibited similar LD blocks, reflecting selection for reproductive traits, while Crioula showed a mixed LD pattern, with strong associations for G3–FecG^EII^ (r^2^ = 0.76). Complete D’ and r^2^ results for the analyzed breeds are provided in Table S3. To visualize allelic correspondence among SNP positions, a multiple alignment of *GDF9* sequences including SNP positions used in the LD analysis was generated (Figure S1).

### 3.4. Coat Color-Related SNPs

**Figure 2:**
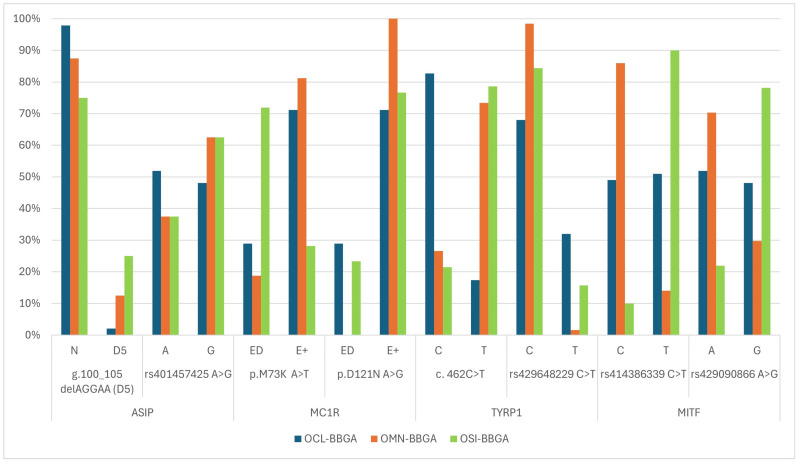
Allelic frequencies (%) of eight SNPs in the *ASIP*, *MC1R*, *TYRP1* and *MITF* genes in ram samples of the Brazilian Animal Germplasm Bank (BBGA), distributed across the breeds: OCL-BBGA—Crioula (N = 26); OMN-BBGA—Morada Nova (N = 32) and OSI-BGGA—Santa Inês (N = 16).

Allele frequency analysis of the *ASIP* gene (chromosome 13: 63,591,538–63,668,259, ovine reference genome ARS-UI_Ramb_v3.0, NC_056066.1) in in situ samples showed that at locus g.100-105del (D_5_), located in exon 2, the 5 bp insertion (N) is the most frequent in most breeds, except for Corriedale, a commercial breed characterized by its white wool, which showed a higher frequency (62%) for the deletion (D_5_) variant (Table 3). In the BBGA sample set, the deletion variant, linked to the dark coat phenotype, was detected at low frequencies in locally adapted Crioula, Morada Nova and Santa Inês (Figure 2).

In the *MC1R gene* (chromosome 14: 14,251,730–14,252,683; ovine reference genome ARS-UI_Ramb_v3.0, NC_056067.1), two SNPs located in exon 1 were analyzed: p.M73K (codon 73) and p.D121N (codon 121). In both these markers, the E^+^ allele was the most prevalent among white-coated breeds, being fixed in Brazilian Bergamasca, Corriedale, Hampshire, Pantaneiro, Suffolk, and Texel. Conversely, dark-coated breeds such as Crioula, Damara, Santa Inês, Dorper, and Brazilian Somali exhibited higher frequencies of the Eᴰ allele, consistent with the well-established dominance pattern between these variants (Table 3). In the BBGA, the E^+^ and E^+^ alleles at variants p.M73K and p.D121N predominated in the Crioula and Morada Nova. However, in the Santa Inês, the Eᴰ allele (p.M73K A>T), associated with darker pigmentation, had a higher frequency (72%), contrasting with 77% of frequency of allele E^+^ in the p.M73K (Figure 2).

In the *TYRP1* gene (chromosome 2: 81,182,552–81,200,943; ovine reference genome ARS-UI_Ramb_v3.0, NC_056055.1), two SNPs were analyzed: c. 462C>T (exon 1) and rs429648229 C>T (exon 2). The C allele at the c.462C>T locus was the most frequent across ten breeds, except for Brazilian Bergamasca, Damara, Morada Nova, Brazilian Fat-tail, and Santa Inês, where the T allele predominated (Table 3). Similarly, Morada Nova and Santa Inês from the BBGA also had allele T as the most frequent (Figure 2). At the rs429648229 C>T locus, the C allele was fixed in Morada Nova and represented the most frequent variant in nearly all breeds conserved in situ, which can also be observed in the BBGA samples (Figure 2). Genotypic frequencies of the eight SNPs located in *ASIP*, *MC1R*, *TYRP1* and *MITF* are available in Table S4.

### 3.5. Haplotypic Frequencies of Coat Color-Related SNPs

Haplotype frequencies of the *MC1R* and *TYRP1* genes were estimated based on the location of SNPs within their respective genes. As previously mentioned, in the *MC1R* gene, the variants analyzed were p.M73K and p.D121N. Three distinct haplotypes were identified: Haplotype 1 (E^+^/E^+^), associated with the white coat phenotype; Haplotype 2 (E^+^/Eᴰ); and Haplotype 3 (Eᴰ/Eᴰ), associated with dark fleece (Table 4).

Haplotype 1 was identified in nearly all breeds, except Dorper, and was fixed in six wool breeds: Brazilian Bergamasca, Corriedale, Hampshire, Pantaneiro, Suffolk, and Texel (Table 4). Haplotype 2 was absent in Brazilian Somali, which was nearly fixed for Haplotype 3 (99%), as well as in Brazilian Bergamasca and Pantaneiro, both fixed for Haplotype 1 (100%). Additionally, Haplotype 2 wasn’t observed in the Crioula, with frequencies split between Haplotypes 1 (49%) and 3 (51%). Haplotype 3 was detected in the Crioula, Dorper, Ile de France, Santa Inês, and Brazilian Somali, but was absent in white-wooled commercial breeds and in the Morada Nova, which, although black-coated individuals are included, does not officially register this phenotype in the breeders’ association.

In the *TYRP1* gene, two SNPs were analyzed: c. 462C>T and rs429648229 C>T, and four haplotypes were identified: Haplotype 4 (C/C), Haplotype 5 (C/T), Haplotype 6 (T/C), and Haplotype 7 (T/T), with Haplotype 4 being the most frequent, present in all genotyped breeds (Table 4).

### 3.6. Association Between Prolificacy and Coat Color

The independence chi-squared test with Monte Carlo simulation revealed statistically significant associations among the analyzed markers. Specifically, the comparison between FecG^E^ and p.M73K A>T yielded X^2^ = 37.95, *p* < 0.001 (Bonferroni-adjusted *p* = 0.0003), P and p.D121N A>G showed X^2^ = 19.56, *p* < 0.001 (adjusted *p* = 0.0012), and the comparison between p.M73K A>T and p.D121N A>G yielded X^2^ = 68.76, *p* < 0.001 (adjusted *p* = 0.0003). These results indicate significant non-random distribution of genotypes among the SNPs.

## 4. Discussion

Following [32], a stringent threshold (>0.90) was applied to genotype calls, as the manufacturer’s recommendation (>0.75) yielded inconsistent heterozygous calls on the Fluidigm platform. Although this excluded several samples and SNPs with low call rates, it reflects reduced reliability at lower thresholds rather than assay failure. These discrepancies were not attributable to primer design quality, as assessed using the D3 design tool; however, prolonged shipping and suboptimal reagent storage conditions in Brazil may have contributed to reduced performance in some reactions. Additionally, several markers were developed initially using European reference genomes, and mismatches caused by population-specific polymorphisms may have impaired primer annealing and, consequently, call rates and QC values in Brazilian breeds.

As expected for the breeds analyzed, a relatively high proportion of monomorphic markers was detected, particularly in the *BMP15* and *BMPR1B* genes. These genes are mainly associated with prolificacy in sheep of European origin. They are not responsible for the trait in Brazilian locally adapted breeds [15], which explains their limited informativeness in our dataset. Given their limited relevance for autochthonous breeds in the country, markers in these genes should be excluded from future studies. Aligned with a previous study [33], the revised panel will retain only informative, non-linked, and consistently performing loci, thereby improving analytical accuracy and overall robustness. This optimization will also facilitate the transfer of specific trait-associated markers (e.g., coat color) to more widely accessible platforms such as real-time PCR, enhancing applicability for routine genotyping.

*GDF9* is an important gene associated with prolificacy in Brazilian sheep. Previously, the FecG^E^ mutation was reported by [13] in Santa Inês animals and posteriorly observed in the Morada Nova breed by [14] and confirmed in both breeds in a subsequent study [34]. Subsequently, the mutation was first reported outside Brazil by [35] in Pelibuey ewes from Mexico, which is also classified as a hair breed. Sheep homozygous for the mutation E/E display a hyperprolific phenotype, with a considerable increase in ovulation rate (82%) and prolificacy (58%) when compared to animals homozygous for the wild type genotype (+/+) [13]. Therefore, FecG^E^ can be strategically used to enhance prolificacy in these hair sheep breeds, as observed in Morada Nova and Santa Inês, considering risks such as elevated inbreeding.

In the present study, in addition to being present in Morada Nova and Santa Inês, the FecG^E^ mutation was found at high frequency in the Brazilian Blackbelly (76%) and at considerable frequencies in the Brazilian Fat-tail (0.43) and the Brazilian Somali (0.30). A previous report documented a high reproductive performance in Brazilian Blackbelly, including the exclusive occurrence of triplet births (3.3%) and the greatest frequency of twins (37%), in contrast to Morada Nova (22%) and Santa Inês (9.5%) [36]. Interestingly, Brazilian Blackbelly is closely related to the Barbados Blackbelly [37], a breed that originated from the Caribbean Sea and historically described as prolific and resilient, despite the lack of empirical support for these traits [38]. Previous investigations in the Barbados Blackbelly reported the absence of Booroola FecB (*BMPR1B*) and FecX^I^ (*BMP15*) mutations [39]. Additionally, the 3′ end regions of the *GDF9* and *BMPR1B* genes were analyzed in Blackbelly from Mexico, but no mutations associated with prolificacy were identified [40].

The present findings therefore offer the first direct evidence on the molecular basis of prolificacy in Brazilian Blackbelly. Furthermore, based on reports in other American hair sheep, such as Santa Inês, Morada Nova, and Pelibuey [13,14,35], we hypothesize that FecG^E^ represents the primary mutation influencing prolificacy across hair breeds of the Neotropics. This hypothesis is reinforced by the exclusive occurrence of the FecG^E^ mutation in locally adapted hair sheep, as most wool breeds genotyped here, including Crioula, lacked the E allele. Only Brazilian Bergamasca and Pantaneiro exhibited low residual frequencies, likely due to historical introgression from Santa Inês to improve meat traits [37,41].

Other mutations evaluated in the *GDF9* gene include G1, G3, and G4, previously genotyped in Cambridge and Belclare sheep, both European breeds [2]. Whereas G1 and G3 are nonfunctional mutations, G4 results in an amino acid substitution, making this variant potentially functional. However, a recent study in Hu sheep found no significant association with prolificacy [42]. In the present study, the wild-type allele A was the most frequent across all Brazilian locally adapted breeds genotyped, suggesting that it does not explain prolificacy in these breeds. Although some of the studied mutations, such as variants 3655, 3776, and rs425223128, showed low polymorphism in most breeds, further research is essential to clarify the role of these and other *GDF9* variants in the prolificacy of Brazilian sheep.

The LD analysis indicated a strong linkage among some *GDF9* variants, which is noteworthy, particularly given the importance of the FecG^E^ mutation for prolificacy in Brazilian hair sheep and its association with rs40376560. The strong LD between these SNPs indicates that rs40376560 can serve as a reliable and cost-effective proxy for FecG^E^ genotyping, facilitating future association studies and marker-assisted selection (MAS), especially given the challenges of designing FecG^E^ allele-specific primers for TaqMan assays. Furthermore, while several marker pairs showed D’ = 1 but low r^2^, they were not interpreted as functionally linked without further validation. Overall, LD patterns differ among breeds, reflecting, in particular, their demographic histories and selective pressures. For example, Morada Nova and Santa Inês exhibit similar LD patterns, likely due to selection for prolificacy.

In the analysis of coat color-related genes, particularly in the *TYRP1* gene, the loci c.462C>T (silent mutation) and rs429648229 C>T (mutation with no known effect) do not alter amino acids and therefore do not affect enzymatic activity [43]. Thus, they are unlikely to explain coat color variation in Brazilian breeds. Consistently, an earlier investigation reported no association between 18 SNPs in *TYRP1* and coat color parameters in Crioula sheep, an important locally adapted wool breed in Brazil [17]. In the *MITF* gene, at the rs414386339 C>T locus, the T allele was the most frequent in commercial breeds, whereas allele C predominated in most autochthonous breeds, such as Brazilian Blackbelly, Brazilian Bergamasca, Morada Nova, Pantaneiro, Brazilian Fat-tail, and Santa Inês, a pattern observed in samples of in situ populations and the BBGA germplasm. Although no functional role has yet been demonstrated for this mutation [8], its potential involvement in the white-body/black-head phenotype warrants further investigation, as the T allele was nearly fixed in Dorper, Damara, Hampshire, and Brazilian Somali, all characterized by this pattern. At the rs429090866 A>G locus, previously associated with coat color variation from white to black [8], the G allele was predominant in most breeds, except in Morada Nova and Brazilian Fat-tail, which include white and red varieties as recognized by ARCO.

**Figure 3:**
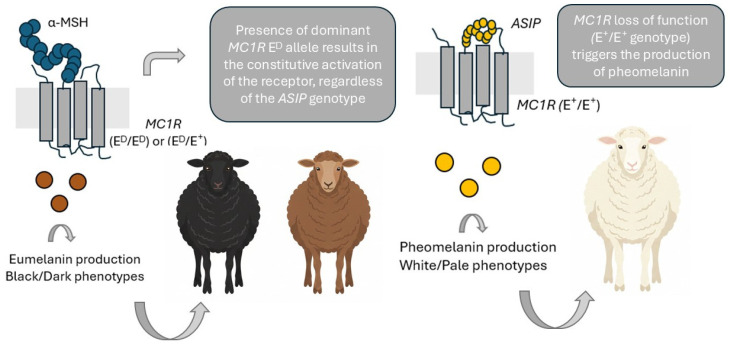
Epistatic interaction between *ASIP* and *MC1R* in sheep pigmentation. The dominant *MC1R* allele (Eᴰ) constitutively activates the receptor and drives eumelanin production regardless of *ASIP* status. *MC1R* loss-of-function (E^+^/E^+^) results in pheomelanin synthesis. Generally, the presence of the *MC1R* wildtype allele is required for the observation of *ASIP* products, demonstrating classical epistasis between the two genes.

Previously, *ASIP* gene variants have been linked to recessive black coat color, particularly the D_5_ deletion [44,45]. However, epistatic interactions between *ASIP* and *MC1R*, reported in several sheep breeds [17,18,46,47,48], may explain the high frequency of D_5_ in Corriedale sheep, where the E^+^/E^+^ alleles at *MC1R* suppress its phenotypic expression. Nevertheless, the 5-bp insertion (N) was the most frequent variant across all breeds, including BBGA samples. A conceptual diagram illustrating the epistatic interaction between *ASIP* and *MC1R* is presented in Figure 3. At the rs401457425 A>G locus, the A allele, dominant for white fleece in Finnsheep [8], was generally less frequent, except in Brazilian Blackbelly, Damara, Brazilian Fat-tail, and Brazilian Somali, all hair sheep breeds. In the woolly Crioula, frequencies were balanced for both alleles in both in situ and ex situ conserved samples. Although linked to white fleece in Finnsheep, further studies are needed to clarify its role in Brazilian breeds and its potential contribution to coat color in hair sheep.

In Crioula sheep, *ASIP* deletions at D_5_ and D_9_ were previously reported to have low homozygous frequencies [18]. However, the present study found a higher proportion of D_5_/D_5_ animals in this breed, likely due to a larger sample size and methodological differences, given the limited reliability of Fluidigm genotyping for large indels, as noted in the D3 manual, available at https://d3.fluidigm.com/ (accessed on 27 November 2025). Although D_5_ and D_9_ deletions are not significantly associated with coat color in Crioula sheep [18], evidence suggests that, together with g.5172T>A and epistatic interactions with *MC1R* alleles (E^+^, Eᴰ), they contribute to the breed’s phenotype [17,18]. However, in the present study, given the reliability issues with the D_5_ indel and the performance problems of g.5172T>A, only the *MC1R* gene SNPs from the reduced panel were used for the Crioula breed, as they already enable reliable inference of the phenotype in this breed.

In the *MC1R* gene, previous studies in Crioula sheep identified the dominant E^D^ allele of variants p.M73K and p.D121N as associated with dark fleece. In contrast, the recessive E^+^ allele is associated with white fleece and occurs only in homozygosity in white individuals [17,49]. Consistently, the present study revealed a predominance of the E^+^/E^+^ haplotype across most analyzed breeds, particularly those with predominantly white coats, in both BBGA and in situ samples. In contrast, E^D^/E^D^ was less frequent and was restricted to Crioula, Dorper, Santa Inês, and Brazilian Somali, breeds that exhibit coat color variation or produce black-headed individuals. Notably, in Crioula sheep, E^D^/E^D^ (51%) and E^+^/E^+^ (49%) were evenly distributed, reflecting the breed’s characteristic phenotypic diversity, with naturally colored fleece ranging from white to black, including gray and brown.

The *MC1R* gene has previously been identified as a determinant of coat color variation in Santa Inês ewes [50]. In this study, all three described haplotypes (E^+^/E^+^, E^+^/Eᴰ, and Eᴰ/Eᴰ) were observed in the breed, consistent with their recognized phenotypic diversity, as Santa Inês has four officially accepted coat color patterns. These findings reinforce the functional association between *MC1R* alleles and coat color expressions in this breed. The dominant E^D^ allele predominates in dark breeds [51,52], whereas it is uncommon in colored animals, suggesting the involvement of other genetic factors in coat color regulation [46,51,52,53,54]. In this study, E^D^ was found at low frequency in the Morada Nova, where only the E^+^/E^+^ and Eᴰ/E^+^ haplotypes were detected, the latter being less frequent. This breed has only two ARCO-recognized varieties, red and white. Black-coated animals, though not uncommon in flocks, are generally excluded from registration [55,56], which may explain the presence of the E^D^ allele in Morada Nova.

Several studies have aimed to elucidate the genetic mechanisms of coat color in small ruminants [57,58,59,60,61,62]. However, in the Brazilian context, research on this topic remains scarce, particularly in hair sheep breeds such as Santa Inês and Morada Nova, where environmental adaptation plays a significant role. It’s worth noting that although the sample sizes were sufficient to capture the genetic variability of the major locally adapted breeds (Santa Inês, Morada Nova, and Crioula), commercial breeds represented by fewer individuals (e.g., Damara, N = 10; Texel, N = 14) may lack rare alleles, which should be considered when interpreting the results. In general, germplasm samples preserved in the BBGA retain the genetic diversity observed in populations conserved in situ, reflecting their representativeness for both traits under study. This indicates the effectiveness of the gene bank in capturing allelic variation in locally adapted populations, which can provide further support for future use of these samples in conservation, breeding, and genomic studies.

A significant non-random distribution of genotypes was observed between prolificacy and coat-color-related loci, indicating a statistical association between the analyzed variants in locally adapted hair sheep. However, these results should be interpreted with caution, as the analyses were restricted to genotypic data only; the phenotypic effects of heterozygous genotypes remain unconfirmed for prolificacy, and coat color determination involves additional, untested loci. Notably, there is no established evidence linking coat color genetics directly to prolificacy in sheep, as most pigmentation studies focus on breed identification, wool, or skin traits rather than reproductive performance. Previous phenotypic studies in Mexican and West African hair sheep [63,64] have suggested potential correlations between coat color and reproduction in some populations, yet these associations are likely influenced by environmental and management factors and do not involve specific genetic markers. In the present study, the observed genotypic associations likely reflect population substructure within the five investigated breeds rather than a direct functional link between prolificacy and coat color. Future studies combining genotypic and phenotypic data are required to accurately assess potential functional relationships between these traits.

This study represents the first genetic screening of prolificacy and coat color-related mutations across a wide range of sheep breeds adapted to Brazilian environments. The results provide previously unavailable information on the distribution and relevance of key functional variants, offering a practical genetic framework for national breeding programs and conservation planning. The reduced SNP panel proved generally effective for identifying mutations of interest, supporting its applicability as a targeted tool for marker-assisted selection. Nonetheless, refinement is warranted, including the exclusion of noninformative variants and reassessment of the Fluidigm platform in favor of more robust genotyping alternatives to address the low call rates observed. Overall, this report addresses a critical genomic gap and provides actionable insights to strengthen selection strategies and the management of locally adapted genetic resources.

Refining the panel also has practical value for breed registration and certification, particularly when coat color is a defining breed attribute. The reduced SNP panel could be further validated for certification purposes, as previously demonstrated for coat color markers in sheep [61,65,66,67]. This would enable the inclusion of individuals not yet officially recognized within their respective breeds, while supporting genetic conservation programs and enhancing the commercial value of animal-derived products. In breeds with strict color standards or regulated pattern requirements, such as Crioula or Morada Nova, inaccurate genotyping of *ASIP* or *MC1R* variants could lead to misclassification, compromising both pedigree authentication and conservation records. An optimized, population-adjusted marker set will therefore support more reliable verification of color-based eligibility criteria and avoid erroneous exclusion or approval of animals in formal registry processes.

## 5. Conclusions

- This report highlights the predominant role of the FecG^E^ (*GDF9*) variant in the prolificacy of Brazilian locally adapted hair sheep, reporting for the first time a high frequency of this mutation in the Brazilian Blackbelly, which provides new insights into the genetic basis of the high prolificacy in this breed.
- Genetic diversity was observed between wool/hair and local/commercial breeds, which exhibited distinct allelic, genotypic, and haplotypic frequencies for the studied markers. This result reflects the phenotypic diversity observed in these breeds and may aid conservation and certification efforts.
- The Brazilian Animal Germplasm Bank seems to retain the genetic diversity found in populations conserved in situ, underscoring its strategic role in conservation efforts.
- The reduced SNP panel effectively genotyped the diversity of Brazilian sheep, confirming which markers are present and segregating in locally adapted breeds. Its applicability could be enhanced by excluding markers of limited relevance.

## Figures and Tables

**Table 1:** Breed description and sample size of in situ and ex situ populations.

Breed	Code	Type	Hair/Wool Color *	Breed Type	Phenotypic Relevance	Sample Size	
						In Situ	Ex Situ
Brazilian Blackbelly	OBN	Hair	Colored	Locally adapted	Dual-Purpose (Meat/Skin)	43	
Brazilian Bergamasca	OB	Wool	White	Locally adapted	Triple-Purpose (Meat, Milk and Wool)	39	2
Corriedale	OC	Wool	White	Commercial	Dual-Purpose (Meat/Wool)	29	
Crioula	OCL	Wool	Colored	Locally adapted	Triple-Purpose (Meat, Wool and Skin)	44	26
Damara	ODA	Hair	White with black head	Commercial	Dual-Purpose (Meat/Skin)	10	
Dorper	ODO	Hair	White with black head	Commercial	Dual-Purpose (Meat/Skin)	18	2
Hampshire	OH	Wool	White	Commercial	Dual-Purpose (Meat/Wool)	14	2
Ile de France	OIF	Wool	White	Commercial	Dual-Purpose (Meat/Wool)	39	
Morada Nova	OMN	Hair	Colored	Locally adapted	Dual-Purpose (Meat/Skin)	129	32
Pantaneiro	OPT	Wool	White	Locally adapted	Triple-Purpose (Meat, Milk and Wool)	46	
Brazilian Fat-tail	ORL	Hair	Colored	Locally adapted	Dual-Purpose (Meat/Skin)	34	
Santa Inês	OSI	Hair	Colored	Locally adapted	Dual-Purpose (Meat/Skin)	233	16
Brazilian Somali	OS	Hair	White with black head	Locally adapted	Dual-Purpose (Meat/Skin)	37	5
Suffolk	OSUF	Wool	White	Commercial	Dual-Purpose (Meat/Wool)	28	
Texel	OT	Wool	White	Commercial	Dual-Purpose (Meat/Wool)	14	3

* Colored animals may include multiple variants (e.g., black, red, and white).

**Table 2:** Allelic Frequencies of 12 prolificacy-related SNPs in the genes *BMP15*, *BMPR1B* and *GDF9* in the main sheep breeds in Brazil.

Gene	Locus	Allele	Breed ^1^														
			OBN	OB	OC	OCL	ODA	ODO	OH	OIF	OMN	OPT	ORL	OSI	OS	OSUF	OT
*BMP15*	B1	CTT	0.62	0.19	0.38	0.59	0.15	0.19	0.82	0.28	0.28	0.42	0.54	0.52	0.37	0.64	0.71
		DEL	0.38	0.81	0.62	0.41	0.85	0.81	0.18	0.72	0.72	0.58	0.46	0.48	0.64	0.36	0.29
*BMPR1B*	G192A	C	0.99	0.96	1	1	1	1	1	0.97	1	0.94	1	0.94	1	1	1
		T	0.01	0.04	0	0	0	0	0	0.03	0	0.07	0	0.06	0	0	0
*GDF9*	G1	G	1	0.95	0.89	1	1	1	1	0.80	0.82	0.99	0.92	1	1	0.82	0.50
		A	0	0.05	0.11	0	0	0	0	0.21	0.18	0.01	0.08	0	0	0.18	0.50
	G3	G	0.97	0.89	0.59	0.63	0.55	0.22	0.57	0.42	0.90	0.65	0.72	0.81	0.73	0.44	0.60
		A	0.03	0.11	0.41	0.37	0.45	0.78	0.43	0.58	0.10	0.35	0.28	0.19	0.27	0.56	0.40
	rs40376560	G	0.79	0.13	0	0	0	0	0	0	0.71	0.07	0.45	0.36	0.30	0	0
		T	0.21	0.87	1	1	1	1	1	1	0.29	0.93	0.55	0.64	0.70	1	1
	G4	G	1	0.99	0.86	1	1	1	1	0.88	1	0.92	1	0.99	1	0.91	0.77
		A	0	0.01	0.14	0	0	0	0	0.13	0	0.08	0	0.01	0	0.09	0.23
	3655	G	1	0.99	0.98	1	0.80	1	0.61	0.95	1	0.97	0.81	0.98	0.88	0.93	0.86
		A	0	0.01	0.02	0	0.20	0	0.39	0.05	0	0.03	0.19	0.02	0.12	0.07	0.14
	3776	G	1	1	1	1	1	1	1	0.95	1	1	0.87	0.99	1	1	1
		C	0	0	0	0	0	0	0	0.05	0	0	0.13	0.01	0	0	0
	FecG^EII^	A	1	0.96	0.74	0.83	0.80	0.47	1	0.61	0.93	0.80	0.94	0.88	0.85	0.70	0.96
		G	0	0.04	0.26	0.17	0.20	0.53	0	0.39	0.07	0.20	0.06	0.12	0.15	0.30	0.04
	FecG^EI^	G	1	0.96	0.76	0.76	0.80	0.47	1	0.60	0.93	0.69	0.94	0.89	0.85	0.70	0.96
		A	0	0.04	0.24	0.24	0.20	0.53	0	0.40	0.08	0.31	0.06	0.11	0.15	0.30	0.04
	FecG^E^	+	0.24	0.87	1	1	1	1	1	1	0.29	0.93	0.57	0.69	0.70	1	1
		E	0.76	0.13	0	0	0	0	0	0	0.71	0.07	0.43	0.31	0.30	0	0
	rs425223128	C	0.99	1	1	0.94	1	1	1	1	1	0.98	1	1	1	1	1
		T	0.01	0	0	0.06	0	0	0	0	0	0.03	0	0	0	0	0

^1^ Breed: OBN—Brazilian Blackbelly (N = 43); OB—Brazilian Bergamasca (N = 39); OC—Corriedale (N = 29); OCL—Crioula (N = 44); ODA—Damara (N = 10); ODO—Dorper (N = 18); OH—Hampshire (N = 14); OIF—Ile de France (N = 39); OMN—Morada Nova (N = 129); OPT—Pantaneiro (N = 46); ORL—Brazilian Fat-tail (N = 34); OSI—Santa Inês (N = 233); OS—Brazilian Somali (N = 37); OSUF—Suffolk (N = 28); OT—Texel (N = 14).

**Table 3:** Allelic frequencies of eight coat color-related SNPs in the genes *ASIP*, *MC1R*, *TYRP1* and *MITF* in the main sheep breeds in Brazil.

Gene	Locus	Allele	Breed ^1^														
			OBN	OB	OC	OCL	ODA	ODO	OH	OIF	OMN	OPT	ORL	OSI	OS	OSUF	OT
*ASIP*	g.100_105 delAGGAA	N	0.99	0.98	0.39	0.61	1	0.96	1	0.88	0.87	0.89	1	0.80	0.98	0.83	1
		D_5_	0.01	0.02	0.61	0.39	0	0.04	0	0.12	0.13	0.11	0	0.20	0.02	0.17	0
	rs401457425 A>G	A	0.52	0.39	0.26	0.44	0.90	0.14	0.07	0.10	0.40	0.41	0.68	0.41	0.69	0.27	0.29
		G	0.48	0.61	0.74	0.56	0.10	0.86	0.93	0.90	0.60	0.59	0.32	0.59	0.31	0.73	0.71
*MC1R*	p.M73K A>T	E^+^	0.94	1	1	0.49	0.30	0	1	0.83	0.73	1	0.85	0.28	0.01	1	1
		E^D^	0.06	0	0	0.51	0.70	1	0	0.17	0.27	0	0.15	0.72	0.99	0	0
	p.D121N A>G	E^+^	1	1	0.96	0.49	1	0.06	1	0.92	1	1	1	0.79	0.01	1	1
		E^D^	0	0	0.04	0.51	0	0.94	0	0.08	0	0	0	0.21	0.99	0	0
*TYRP1*	c. 462C>T	C	0.26	0.34	0.79	0.75	0.42	0.83	0.57	0.69	0.29	0.67	0.20	0.25	0.70	0.70	0.96
		T	0.74	0.66	0.21	0.25	0.58	0.17	0.43	0.31	0.71	0.33	0.80	0.75	0.30	0.30	0.04
	rs429648229 C>T	C	0.79	0.87	0.62	0.67	0.50	0.61	0.43	0.90	1	0.74	0.93	0.92	0.40	0.64	0.93
		T	0.21	0.13	0.38	0.33	0.50	0.39	0.57	0.10	0	0.26	0.07	0.08	0.60	0.36	0.07
*MITF*	rs414386339 C>T	C	0.88	0.72	0.40	0.49	0.10	0.10	0.14	0.36	0.86	0.58	0.74	0.80	0.10	0.39	0.29
		T	0.12	0.28	0.60	0.51	0.90	0.90	0.86	0.64	0.14	0.42	0.26	0.20	0.90	0.61	0.71
	rs429090866 A>G	A	0.50	0.27	0.05	0.48	0.30	0.28	0.25	0.40	0.80	0.34	0.63	0.29	0.50	0.34	0.25
		G	0.50	0.73	0.95	0.52	0.70	0.72	0.75	0.60	0.20	0.66	0.37	0.71	0.50	0.66	0.75

^1^ Breed: OBN—Brazilian Blackbelly (N = 43); OB—Brazilian Bergamasca (N = 39); OC—Corriedale (N = 29); OCL—Crioula (N = 44); ODA—Damara (N = 10); ODO—Dorper (N = 18); OH—Hampshire (N = 14); OIF—Ile de France (N = 39); OMN—Morada Nova (N = 129); OPT—Pantaneiro (N = 46); ORL—Brazilian Fat-tail (N = 34); OSI—Santa Inês (N = 233); OS—Brazilian Somali (N = 37); OSUF—Suffolk (N = 28); OT—Texel (N = 14).

**Table 4:** Estimated haplotypic frequencies based on two SNPs in the *MC1R* gene and two SNPs in the *TYRP1* gene across Brazilian sheep breeds.

Breed ^1^	N	*MC1R*			*TYRP1*			
		H1	H2	H3	H4	H5	H6	H7
		(*E^+^/E^+^*)	(*E^+^*/*E^D^*)	(*E^D^/E^D^*)	(C/C)	(C/T)	(T/C)	(T/T)
OBN	43	0.94	0.06		0.22	0.03	0.58	0.17
OB	39	1			0.34		0.53	0.13
OC	29	1			0.52	0.27	0.17	0.04
OCL	44	0.49		0.51	0.50	0.25	0.17	0.08
ODA	10	0.30	0.70		0.12	0.30	0.22	0.36
ODO	18			1	0.55	0.28	0.06	0.11
OH	14	1			0.22	0.35	0.21	0.22
OIF	39	0.83	0.09	0.09	0.60	0.09	0.29	0.02
OMN	129	0.73	0.27		0.28	0.01	0.71	
OPT	46	1			0.52	0.15	0.22	0.11
ORL	34	0.85	0.15		0.18	0.02	0.77	0.03
OSI	233	0.28	0.52	0.20	0.22	0.03	0.70	0.05
OS	14	0.01		0.99	0.27	0.43	0.17	0.13
OSUF	37	1			0.36	0.35	0.26	0.03
OT	28	1			0.92	0.04		0.04

^1^ Breed: OBN—Brazilian Blackbelly (N = 43); OB—Brazilian Bergamasca (N = 39); OC—Corriedale (N = 29); OCL—Crioula (N = 44); ODA—Damara (N = 10); ODO—Dorper (N = 18); OH—Hampshire (N = 14); OIF—Ile de France (N = 39); OMN—Morada Nova (N = 129); OPT—Pantaneiro (N = 46); ORL—Brazilian Fat-tail (N = 34); OSI—Santa Inês (N = 233); OS—Brazilian Somali (N = 37); OSUF—Suffolk (N = 28); OT—Texel (N = 14).

## Data Availability

Data available on request due to restrictions.

## Supplementary Materials

The following supporting information can be downloaded at: https://www.mdpi.com/article/10.3390/ani16132008/s1, Table S1: Prolificacy and Coat Color-associated markers genotyped in the genes BMPR1B, BMP15, GDF9, ASIP, MC1R, TYRP1 and MITF; Table S2: Genotypic frequencies of 12 SNPs located in BMP15, BMPR1B and GDF9 genes in Brazilian sheep; Table S3: D’ and r2 estimates of linkage disequilibrium among 10 GDF9 SNPs in Brazilian sheep breeds; Figure S1: Multiple sequence alignment of GDF9 gene highlighting the positions of each marker analyzed for linkage disequilibrium; Table S4: Genotypic frequencies of eight SNPs located in the genes *ASIP*, *MC1R*, *TYRP1* and *MITF* in the main sheep breeds of Brazil. References [2,8,43,45,51,68,69,70,71,72,73] are cited in the supplementary materials.
