# Supplementary figures and images for: Assessment of a Reduced SNP Panel Targeting Prolificacy and Coat Color Genes in Brazilian Sheep Breeds
Source: Animals (Basel). 2026 Jul 1;16(13):2008. doi: 10.3390/ani16132008 (PMC13359520; doi:10.3390/ani16132008)

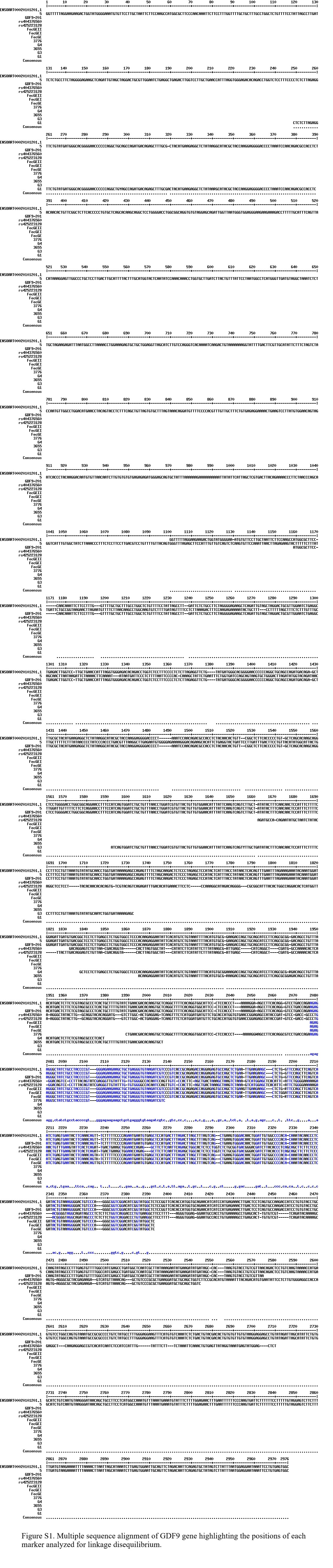

Supplement: Supplementary file 1 [file animals-16-02008-s001.zip › Figure S1.jpg]
